# The effects of combined motor control and isolated extensor strengthening versus general exercise on paraspinal muscle morphology and function in patients with chronic low back pain: a randomised controlled trial protocol

**DOI:** 10.1186/s12891-021-04346-x

**Published:** 2021-05-22

**Authors:** Maryse Fortin, Meaghan Rye, Alexa Roussac, Neda Naghdi, Luciana Gazzi Macedo, Geoffrey Dover, James M. Elliott, Richard DeMont, Michael H. Weber, Véronique Pepin

**Affiliations:** 1 Department Health Kinesiology and Applied Physiology, Concordia University, 7141 Sherbrooke Street W, SP-165.29, Montreal, Quebec H4B 1R6 Canada; 2 PERFORM Centre, Concordia University, Montreal, Quebec Canada; 3 Centre de Recherche Interdisciplinaire en Réadaptation (CRIR), Montreal, Quebec Canada; 4 School of Rehabilitation Science, McMaster University, Hamilton, Ontario Canada; 5 Department of Physical Therapy and Human Movement Sciences, Feinberg School of Medicine, Northwestern University, Chicago, IL USA; 6 Northern Sydney Local Health District, The Kolling Institute and Faculty of Health Sciences, The University of Sydney, Sydney, NSW Australia; 7 Department of Orthopedic Surgery, McGill University Health Centre, Montreal, Quebec Canada

**Keywords:** Low back pain, Motor control, Multifidus, MRI, Ultrasound

## Abstract

**Background:**

Exercise is a common approach for the management of patients with chronic non-specific low back pain (LBP). However, there is no clear mechanistic evidence or consensus on what type of exercise is more effective than others. While considerable evidence suggests a link between lumbar muscle health (e.g., atrophy and fatty infiltration) with functional deficits, it is unknown whether exercises targeting the lumbar spine can lead to noticeable improvements in muscle health and functional outcomes. The primary aim of this study is to compare the effect of combined motor control and isolated strengthening lumbar exercises (MC + ILEX) versus a general exercise group (GE) on multifidus muscle morphology (size and composition). Secondary aims include assessing the effect of the interventions on overall paraspinal muscle health, pain and disability, as well as psychological factors as possible effect modifiers.

**Methods:**

A total of 50 participants with chronic non-specific LBP and moderate to severe disability, aged between 18 and 60, will be recruited from the local orthopaedic clinics and university community. Participants will be randomised (1:1) to either the MC + ILEX or GE group. Participants will undergo 24 individually supervised exercise sessions over a 12-week period. The primary outcome will be multifidus morphology (atrophy) and composition (fatty infiltration). Secondary outcomes will be muscle function (e.g., % thickness change during contraction), morphology, lumbar extension strength, pain intensity and disability. Potential treatment effect modifiers including maladaptive cognitions (fear of movement, catastrophizing), anxiety, depression, physical activity, and sleep quality will also be assessed. All measurements will be obtained at baseline, 6-week and 12-week; self-reported outcomes will also be collected at 24-week. Between-subjects repeated measure analysis of variance will be used to examine the changes in paraspinal muscle morphology over the different time points. Linear mixed models will be used to assess whether baseline scores can modify the response to the exercise therapy treatment.

**Discussion:**

The results of this study will help clarify which of these two common interventions promote better results in terms of overall paraspinal muscle heath, back pain, disability and psychological factors in adults with chronic LBP.

**Trial registration:**

NTCT04257253, registered prospectively on February 5, 2020.

## Background

Low back pain (LBP) is one of the leading causes of disability worldwide [1, 2], costing billions of dollars in Canada alone each year, including health care costs and missed work [3]. While chronic LBP is a prevalent and persistent global burden, up to 90% of the North American population alone, is at risk of developing LBP [3–5]. Of these cases, only 10% are thought to be the result of identifiable radiological characteristics, such as nerve root compression, fractures, or stenosis [6]. The remaining cases are classified as non-specific LBP, which unsurprisingly, present with a variety of clinical manifestations without diagnostic or therapeutic options. LBP is significantly associated with high levels of disability, decreased function and participation in life as well as decreased sleep quality and increased depressive symptoms [7–9]. Currently, exercise therapy is the most common conservative treatment for LBP as it is easily accessible and can be individually tailored to patient’s needs [10]. Exercise has been bound to improve pain, quality of life and psychosocial aspects of pain such as pain-related fear (catastrophizing), kinesiophobia, depression, and anxiety [11–13]. Although exercise is more effective than no intervention, the effect size of exercise are small to moderate, and to date there is no evidence that one exercise approach is superior to another [14–16].

Individuals with LBP are more likely to have muscle atrophy, fatty infiltration, and asymmetries of the lumbar musculature, specifically the multifidus muscle [17–19]. Possible mechanisms for degenerative muscle changes include poor motor control, decreased muscle activation, and fatigue. Loss of motor coordination can lead to repetitive mechanical stress on surrounding structures, increasing the risk of instability, joint overloading, and pain [20]. Therefore, exercise interventions usually target the lumbar paraspinal muscles specifically [15, 16].

There is mixed evidence as to whether current exercise interventions actually results in morphological changes in the paraspinal muscles (e.g., hypertrophy, reversal of fatty infiltration) and whether paraspinal muscle physiological adaptations will translate in better improvement in patient-related outcomes [21–24]. A recent preliminary study examined the effect of a resistance-based exercise protocol on the cross-sectional area (CSA) and fatty infiltration for both the erector spinae and multifidus muscles [22]. The intervention did not yield any significant morphological change but the investigators suggested further exploration into identifying what types of resistance exercise works best. While another recent study reported decreased in paraspinal muscle fatty infiltration following a 16-week free-weight-based resistance training intervention without isolated lumbar extension exercise [23]. In recent years specific motor control interventions have gained popularity given its focus on retraining control of trunk muscles activation, alignment and movement in order to restore proper function [16]. Motor control exercises often target paraspinal muscles directly however, there is limited evidence on their effectiveness in improving spinal muscle morphology [21, 22].

Previous studies that have used targeted strengthening exercises to improve lumbar muscle strength recommend using exercises that include increased pelvic restraints which limit the activity of other large muscles of the posterior chain (e.g., gluteus and hamstrings) [15, 25]. Since most studies do not include exercises where the pelvis is stabilized it is possible that the lack of morphological changes observed in spinal muscles may be due to the compensation of the other posterior chain muscles [25]. Further studies are needed to examine whether pelvic stabilization during extension exercises is able to effectively target the lumbar extensors and lead to significant morphological and functional changes. Furthermore, we are also aware of only one previous study that has examined the correlation between changes in lumbar muscle morphology with fear avoidance and psychosocial factors [22]. High fear avoidance in patients with chronic LBP is significantly associated with less improvement in disability after 1 year as well as longer sick leave [26]. As such, controlling for factors that could influence the change in disability is crucial.

There is limited evidence linking the physiological and morphological changes in paraspinal muscles with decreases in LBP and self-reported improvements in pain and daily function. Therefore, the primary objective of this study is to compare the effects of combined motor control and isolated lumbar strengthening exercises (MC + ILEX; targeted exercise intervention) versus those of a general exercise group (GE: control intervention) multifidus muscle morphology (e.g. size, fatty infiltration). The secondary aim is to compare the effect of each intervention on overall paraspinal muscle health (morphology and function), and the association of these changes with pain and disability. An exploratory aim is to investigate if psychological factors (e.g., kinesiophobia, catastrophizing, anxiety, depression and sleep) can modify the response to the exercise interventions.

## Methods/design

### Study design

**Fig. 1:**
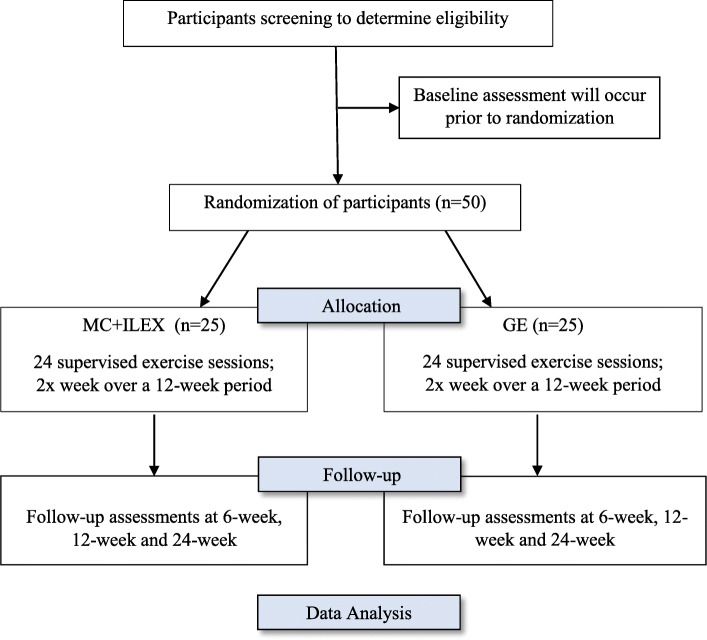
Consort flow diagram

The proposed study is a two-arm prospective randomized controlled trial (RCT), with test-retest design (Fig. 1). This protocol was reported in accordance with the SPIRIT guidelines [27] and CERT recommendation for Exercise Interventional Trials [28].

### Study setting

This study will be conducted at the PERFORM Centre, Concordia University (registration trial NTCT04257253). The proposed project was approved by the Central Ethics Research Committee of the Quebec Minister of Health and Social Services (# CCER-19-20-09). All participants will sign a consent form prior to entering the study.

### Participant recruitment

Participants will be recruited by clinicians in local orthopaedic clinics working in Montreal, Canada and from the local university community by email advertising. Clinicians will assess the participant for eligibility and send referral to the research team, which will confirm eligibility. Similarly, a member of the research team will interview individuals who respond to the advertisement to confirm eligibility.

### Participants

#### Inclusion criteria

Participants must meet all of the following criteria for inclusion:

- Chronic, non-specific low back pain (LBP) for a minimum of 3 months (with or without leg pain)
- Aged between 18 and 65 years old
- Speak either French or English
- Currently seeking care for LBP
- Score either “moderate” or “severe” on the modified Oswestry Low Back Pain Questionnaire
- Do not engage in any sport or fitness training specifically for the lower back muscles up to 3 months before the start of the trial

#### Exclusion criteria

Participants will be excluded if they meet one of the following criteria:

- Any evidence of nerve root compression or reflex motor sign deficits.
- Previous spinal surgery or vertebral fractures.
- Major lumbar spine structural abnormalities (e.g., spondylosis, spondylolisthesis, scoliosis > 10°)
- Pregnancy
- Health conditions that prevent the safe participation in physical exercise as determined by the Physical Activity Readiness Questionnaire.

### Randomization

Participants will be randomly assigned to treatment groups (1:1) using consecutively numbered sealed opaque envelopes (e.g. computer-generated randomization sequence with permuted blocks) created by an individual not involved in the study.

### Blinding

Only the assessor will be blinded, as blinding of therapists and participants is generally not possible in exercise intervention trials [29].

### Procedure

All research activities will take place at the PERFORM Centre, Concordia University. This centre houses 8000m^2^ of laboratories, assessment suites, and lifestyle intervention space, all designed specifically to foster interdisciplinary environment devoted to health prevention research. The infrastructure has all the necessary research platforms (e.g., Imaging Suite, Conditioning floor and Functional Assessment suite) for the conduction of the proposed RCT.

Eligible participants will be randomized between the GE (*n* = 25) and MC + ILEX (n = 25) groups. The participants of the two groups will undergo a 12-week intervention programme with two individually supervised exercise sessions per week of approximately 45 min. The treatment will be provided by a certified athletic therapist (MC + ILEX) with 2 y of clinical experience or graduate exercise science master’s student (GE). Participants in both groups will be advised to complete a home exercise programme during the intervention and after discharge. During the intervention period, participants will be asked to refrain from receiving other types of treatment (e.g., chiropractor, osteopath, massage) and medication, although this will not hinder participation. Participants will be asked to report any co-interventions.

### General exercise group (GE)

The GE participants will engage in a program that will include a 10-min aerobic warm-up (incline treadmill walking or stationary bike), resistance training exercises, followed by trunk-leg stretches. The machine-based resistance training program will be divided into a 2-day split (non-consecutive days) with different muscle group focuses for each day (Table 1). Three sets will be performed for each exercise. The difficulty of the intervention will be progressively increased over the course of the 12-week intervention based on a study procedure developed by Iversen et al. [30] with the following target repetitions: week 1–2, 15–20 repetitions; week 3–5, 12–15 repetitions, week 6–8, 10–12 repetitions; week 9–12, 8–10 repetitions. The weights will be increased by 5% as soon as the participants are able to complete 2 more repetitions than the amount assigned for that period. Stretching exercises will be performed at the end of each session (e.g., cat cow, pigeon, deep lunge, piriformis stretch) and each position will be held for 10 s, 3 times on each side in accordance with ACSM guidelines. As most patients with LBP sedentary lifestyles due to the nature of their pain, the overall goal of this intervention is to return patients to the normal activities of daily living (e.g., rising, bending, lifting, walking) by enhancing lower-body strength and flexibility. Such general exercise programs have been found to reduce pain and improve function (e.g., moderate level of evidence) [31].

**Table 1:** Two-day split exercise program performed by the GE group

Day 1	Day 2
Hip extension (multi-hip machine)*	Goblet Squat
Prone Leg Curl*	Step up
Lat pull down*	Leg extension*
Seated row*	Peck deck*
Hip Abduction*	Lying side hip raises
Hip Abbuction*	Abdominal curl

*Exercise performed on a resistance machine

### Combined motor control and isolated lumbar extension resistance training (MC + ILEX)

The degenerative changes seen in the muscles of patients with LBP can be caused by poor motor control, which can lead to repetitive mechanical stress on surrounding structures, increasing the risk of instability, joint overloading, and pain [20]. Therefore, this intervention will consist of exercises that directly target the deep lumbar muscles with an overall aim of restoring proper coordination, control and co-contraction of these muscles to support the spine at rest and during movement [32, 33]. The intervention will follow principles of motor control and will include a cognitive phase (e.g. activation of the deep spinal muscles) and will transition to an associative and autonomous phase (e.g. functional rehabilitation) [34, 35]. The intervention will also include the coordination and optimal control of global trunk muscles [36, 37].

#### Phase 1: cognitive phase

The initial phase starts with an assessment of muscle activation and breathing patterns. The motor control program will be guided by deficiencies found during the assessment. The primary focus of the intervention is on the correction of the muscle patterns such as increasing the activation of the deep trunk muscles (e.g., multifidus and transverse abdominus muscles) and decreasing the activity of the global muscles (Table 2) [33, 36, 37]. Activation of the deep trunk muscles will be completed in a variety of starting positions that will be progressed in complexity as the patient improves. Before progressing to stage two the patient must be able to meet the following criteria: complete 10 reps while holding for 10 s, achieving activation with minimal feedback or cues, and be able to maintain a normal breathing pattern throughout the exercises [38].

**Table 2:** Sample cues and positions for early Multifidus and Transerse Abdominis activation

Multifidus Activation	
Positions	Prone or on hands and knees (some people are better in 1 to start)
	Fingers on either side of spinous process; evaluation of different spinal levels from T1/T2 to T5/S1
Cues	Try to swell muscle up into my fingers
	Think about tilting pelvis without actually doing it
	Imagine tensing a cable from your pelvis up through your spine
Ideal Response	Symmetrical contraction
	No global muscle activation
	Normal breathing
	Able to hold 10 x 10s
**Transverse Abdominis Activation**	
Positions	Start supine or crook-lying
	Find neutral pelvis
	Place fingers slightly medial and inferior to ASIS
Cues	Try to pull your belly button down to the table
	Try to move your fingers together (medially)
Ideal Response	Gradual increase in tension; 10–15% effort
	Symmetrical contraction
	No global muscle activation
	Normal breathing
	Able to hold 10 x 10s

Breathing will be assessed supine and in sitting for asymmetry, expansibility and for the use of accessory muscles (e.g., sternocleidomastoid and scalene). Correction of breathing patterns with a focus on diaphragmatic breathing will be incorporated into the exercises of both phases of the intervention.

#### Phase 2

Once the patient is able to adequately activate the deep trunk muscles with minimal compensation of the superficial muscles, while maintaining proper breathing, participants will progress to phase 2. This will include the addition of loads to the muscles first into static positions and eventually into dynamic positions [37]. During this phase, the exercise will progress towards functional activities while maintaining lumbar position and coordination of the deep trunk muscles. Exercise difficulty and progression will be achieved by moving the participant into a more challenging position (e.g., supine to sitting), increasing the load (movement of limbs), and introducing the need for dynamic stability (moving to unstable surface such as sitting on an exercise ball). The goal of this phase is to progress to automatic activation of deep trunk muscle with coordination of superficial muscles.

**Fig. 2:**
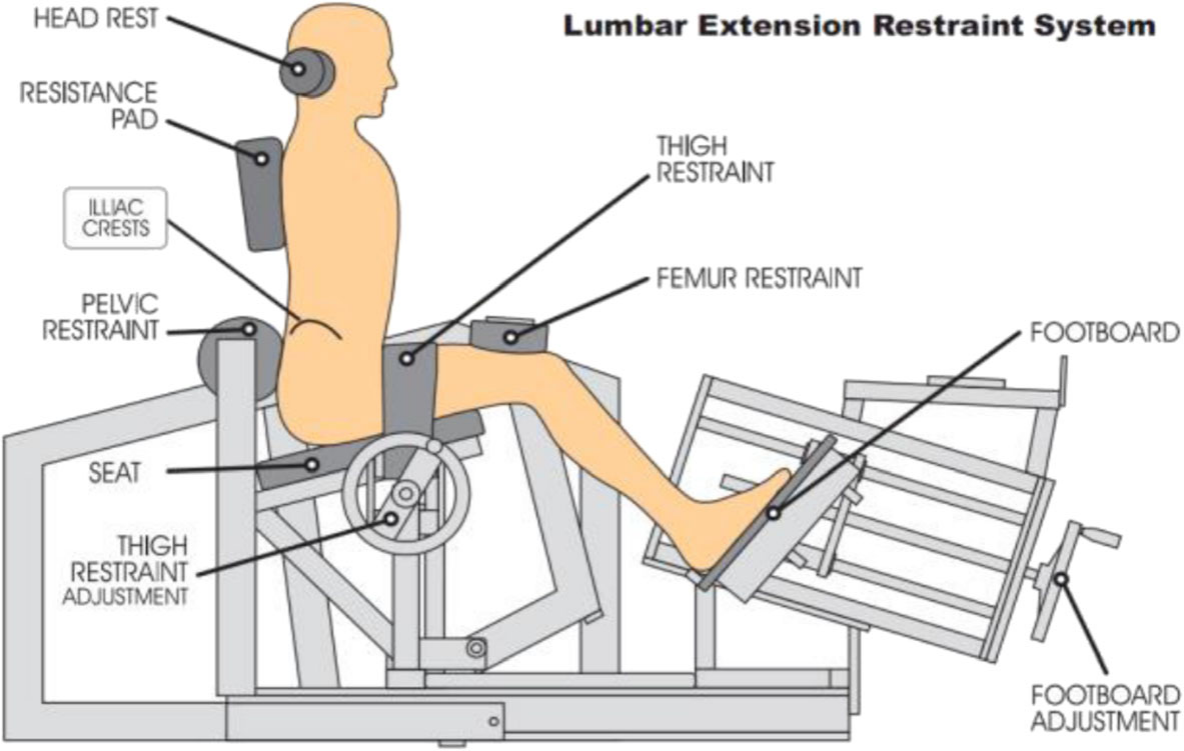
Schematic illustration of the MedX Lumbar medical machine. Image reprinted with permission [41]

Participants in this group will also complete isolated lumbar extensor strength exercise (ILEX) in parallel to the motor control (MC) exercises. This training will be completed on the MedX machine (Fig. 2). Participants’ one repetition maximum (1RM) will have been recorded during the baseline testing (refer to section below about baseline extensor strength assessment). They will perform 2 sets of 15–20 repetitions of lumbar extension at 55% of their baseline 1RM at 24°. Progressions will occur once the patient is able to complete 15–20 repetitions. For each progression the load will be increased by 5% [39, 40]. The MedX lumbar extensor machine, shown in Fig. 2, allows for isolated testing and strengthening of the lumbar extensor muscles through full range of motion in the flexion-extension plane of movement. Pelvic stabilization and lower body restraints eliminate the activation of synergistic and compensatory muscles, such as the glutes and hamstrings, allowing for isolated lumbar extensor strengthening.

### Data collection

All outcomes (as described further) will be obtained at baseline, 6-week and 12-week for both intervention groups. The self-reported outcomes will also be assessed 24-week post-intervention. All self-reported questionnaires will be completed either in-person using paper forms or through the LORIS system using a URL link. Imaging outcomes (e.g., MRI and ultrasound) and lumbar extensor muscle assessments (e.g., strength) will be obtained at the PERFORM Centre, Concordia University. Demographic characteristics will be obtained via a self-reported questionnaire at baseline, after the randomization.

### Outcome measures

#### Primary outcome

##### Multifidus muscle morphology

Multifidus muscle size and fatty infiltration (e.g., from L1 to L5) will be examined using T2-weighted and IDEAL (lava-flex, 2-echo) sequences obtained using a 3-Tesla GE MRI machine. The cross-sectional area (CSA) of the muscle will be taken on axial T2-weighted slices from the cranial view of L1 and the caudal view of L5; single level and total left and right 3D volume will be calculated. The overall area of lean muscle mass will be assessed using a highly reliable thresholding technique [42, 43], and DIXON axial water and fat images will be used to assess percent-fat signal fraction at each spinal level [44]:

$$ \%\mathrm{FSF}=\left({\mathrm{Signal}}_{\mathrm{fat}}/\left[{\mathrm{Signal}}_{\mathrm{water}}+{\mathrm{Signal}}_{\mathrm{Fat}}\right]\times 100\right). $$

#### Secondary outcomes

##### Multifidus muscle function

Multifidus muscle thickness at rest and during submaximal contraction will be evaluated by examining the changes in the muscle during contralateral arm lifts. Muscle thickness will be assessed using the Logic E GE ultrasound machine with a 5-MHz transducer. The submaximal and maximal contractions will be performed 3 times on each side in a prone position. Submaximal contraction will be assessed by instructing the participant to lift their arm while holding a handheld weight (e.g. based on subject’s body weight) while the evaluator examines the contralateral multifidus using the ultrasound [45, 46]. The thickness change in the multifidus muscle between sub-maximal (rest) and maximal (contracted) will be calculated using the following equation: %thickness change = (thickness_contracted_ − thickness_rest_)/thickness_rest_) × 100. This method of assessing multifidus using ultrasound is both reliable and valid as demonstrated by previous experiments [45, 47, 48].

##### Lumbar extensor muscle strength

Lumbar extensor muscle strength will be assessed with the use of the MedX lumbar extension machine. Participants’ hips, knees, and pelvis will be secured to the machine ensuring isolation of the lumbar extensor muscles with the axis of movement being fixed between vertebral levels L5-S1. This dynamometer assesses isometric lumbar extension muscular strength (torque) in a seated position and accommodates the dynamic resistance through a full 72° range of motion (ROM). Therefore, maximum lumbar extension torque will be assessed as maximum voluntary isometric contraction (MVIC) in lumbar extensor muscle strength in seven positions: 72°, 60°, 48°, 36°, 24°, 12° and 0° of flexion [41, 49]. Participants will be seated and positioned in the equipment; initial testing will be performed to verify any limitations in their ROM and adjustment for the counterweight [48]. Participants will first perform a slow controlled warm up for ~ 1 min, and then the maximum strength test will begin [41]. Verbal encouragement will be provided to encourage participants to generate maximum torque. The movement arm of the MedX machine is attached to a load cell that is interfaced with a computer, what will record and calculate torque measurements.

##### Disability

The Oswestry Disability Index (ODI) will be used to measure participants’ level of self-reported disability in relation to LBP. It is a 10-item scale in which each item is rated from 0 to 5, where 0 means that their pain does not influence that situation and a score of 5 indicates severe disability. The categories included in the questionnaire are pain, walking, lifting, sitting, standing, personal care, sleeping, travel, sex life, and social life. Scores are categorized as minimal, moderate, severe, crippled, or bed bound. The ODI has shown good reliability and validity, and therefore is considered to be the gold standard of measuring disability related to low back pain [50].

##### Health related quality of life

The 12-item Short Form Health Survey (SF-12) is the condensed form of the previous 36-item survey and will be used to assess participants’ health-related quality of life. The 12-item survey consists of 8 domains that assess both physical and mental components of health: 1) limitations in physical activities because of health problems, 2) limitations in social activities because of physical or emotional problems, 3) limitations in usual role activities because of physical health problems, 4) bodily pain, 5) general mental health (psychological distress and well-being), 6) limitations in usual role activities because of emotional problems, 7) vitality (energy and fatigue) and 8) general health perceptions. Scores from each of the 12 questions are combined to give an overall score between 0 and 100, with a score of 100 indicating the highest level of health. Given that this is a condensed version of a longer and established questionnaire, it has been extensively tested and shown to be both reliable and valid [51, 52].

##### Pain

The Visual Numerical pain rating scale (NPR) will be used to assess participants’ level of pain. The NRP is a self-reported rating system for pain intensity. Ratings range from 0 to 10 with 0 being no pain, 1–3 being mild pain, 4–7 being moderate pain, and 8–10 being extreme pain. This scale has excellent reliability and validity, and can be used detect statistical and clinically significant changes in perceived pain [53, 54].

### Possible effect modifiers

#### Catastrophizing: pain Catastrophizing scale (PCS)

The PCS is a 13-item questionnaire and will be used to assess participants’ level of catastrophizing. Each item is rated from 0 to 4 for a possible total of 52. The questionnaire focuses on three domains that have been used to describe catastrophizing: attentional focus on pain related thoughts (rumination), exaggeration of painful stimuli (magnification), and adopting a hopeless orientation with coping (helplessness). The higher the score, the higher the level of catastrophizing, with scores above 30 being clinically significant. This scale is both reliable and valid [55, 56].

#### Kinesiophobia: Tampa scale of Kinesiophobia (TSK)

The TSK will be used to measure participants’ fear of movement or reinjury in the presence of pain. The TSK-11 contains 11 phrases related to kinesiophobia, such as “I’m afraid I might injure myself if I exercise”, with each rating as a Likert scale from 1 to 4. The scores range between 11 and 44 with increasing scores showing increased levels of kinesiophobia. This tool has been shown to have high reliability and validity [57].

#### Depression: hospital anxiety and depression scale (HADS)

The HADS is a 14-item questionnaire and will be used to assess participants’ level of depression and anxiety. Seven items are related to depression while the other 7 relate to anxiety. Cognitive, behavioural, and emotional symptoms are covered in the questionnaire. Each item is rated from 0 to 3 with either depression or anxiety having scores between 0 and 21, with 21 being the highest level possible. Scores for either domain between 0 and 7 are classified as normal, 8 to 10 as borderline, and 11 to 21 as abnormal or elevated. The HADS was found to be both reliable and valid [58].

#### Sleep: insomnia severity index (ISI)

The ISI will be used to assess self-reported quality of sleep. It contains 7 questions than consider the ability to fall asleep, the ability to stay asleep, and effects on daily life. Each question is rated with a Likert-scale from 0 to 4, with lower ratings indicating a higher quality of sleep. Scores between 0 and 7 indicating no clinically significant insomnia, 8 to 14 indicating subthreshold insomnia, 15 to 21 indicating moderate insomnia, and 22 to 28 indicating severe insomnia. Fourteen has been commonly used as the cut-off score to detect primary insomnia. The reliability and validity of this tool has been demonstrated [59].

#### Physical activity: international physical activity questionnaire (IPAQ)

The IPAQ will be used to assess participants’ level of physical activity. The IPAQ is a self-reported log of physical activity (METs based on intensity) in minutes per week over a span of 7 days. The level of physical activity is rated either vigorous (8 MET), moderate (4 MET), walking (3.3 MET) and sitting/rest (1 MET) and must be assigned to the right category. The number of minutes per category is then added up and results are classified as high, moderate, or low physical activity based on the total MET minutes. This measure has been deemed both reliable and valid [60].

### Data monitoring

#### Adverse events

The occurrence of adverse events (e.g., muscle soreness and temporary increase in LBP) will be monitored by the therapists during the intervention and at the end of the intervention using open ended questions.

#### Adherence

Adherence with the exercise program will be assessed and noted by the therapists using the participants treatment files. At the end of the intervention, the adherence with home exercises program after discharge will be measured using the following ordinal scale; How often did you perform home exercises? 1) none of the time, 2) some of the time, 3) most of the time, 4) almost all of the time, 5) all of the time) [61].

#### Co-interventions

Participants will be asked to report any type of co-interventions (e.g., osteopathy, chiropractic, massage therapy) during the intervention period at the end of the study. Pain medication will be allowed as it would be unethical to withhold medication, but this information will be collected for all participants.

#### Data integrity

The database will be saved and maintained on a secured network at the institution. Any inconsistencies in the data will be reported, explored and resolved. Only study personnel will have access to the password-protected database. Investigators will allow verification of the data from the ethics board and maintain adequate and accurate records of all documents. Any modifications to the protocol will be reported and communicated to the REB. Confidentiality of the data will be protected and maintained during and after the trial. Data will be stored at Concordia University for a minimum of 5 years after the study closure.

### Sample size calculation

Sample size calculation was determined by using the effect size (e.g., significant pre-post difference in multifidus muscle CSA measurements following a motor-control exercise intervention) from a previous study^62^ (e.g., previously reported effect size (95% CI) for each spinal level; L2 = 0.87(0.20,1.54); L3 = 0.90 (0.19,1.62); L4 = 1.00(0.32, 1.67), and L5 = 0.81(0.10,1.53)) [62]. The mean effect size (over the 4 different spinal levels) was calculated and used for sample size estimation. Pre-post results were considered as independent (independent from two groups) to concur with the present study statistical analysis (between-group factor). Therefore, sample size estimation was calculated with the G*power software (mean difference, independent t-test) on the basis of a mean effect size d = 0.90, 80% power and a significance level of alpha 0.05, and allowing for a 10% lost to follow-up and 10% treatment non-adherence.

### Statistical analysis

Descriptive statistics for demographic characteristics and outcomes will be calculated. The pre to post intervention changes in primary and secondary measures will be evaluated with the use of a between-subjects repeated measures ANOVA. Linear mixed models will be used to assess whether baseline psychological scores can modify the response to the exercise therapy treatment. A *p*-value of < 0.05 will be considered statistically significant.

### Dissemination of findings

The results of this trial will be published in peer-reviewed journals and presented at conferences. After publication of manuscripts, data request can be submitted to the principal investigator (MF).

## Discussion

LBP is one of the most disabling diseases and a major health issue. In accordance with the ever-increasing body of evidence demonstrating accelerate atrophy and fatty infiltration in patients with chronic LBP, improving strength, function and control of the trunk muscle through therapeutic exercises is a primary goal in physical rehabilitation of patients with LBP. As such, strengthening, lumbar stability, and motor control exercises are recommended and amongst the most popular exercises for the management of chronic LBP [14–16, 63]. If specific targeted lumbar muscle exercises are to be prescribed and used clinically, the evaluation of physiological muscle changes, such as hypertrophy and reversal of fatty infiltration and whether they mediate improvements in functional status should be considered when assessing the effectiveness of different exercise interventions. This trial aims to investigate whether MC + ILEX has any superiority to GE in improving overall paraspinal muscle health and related clinical symptoms. Our research protocol through measurement of muscle morphology and function, clinical symptoms and psychological factors will shed some light into this field. The results of this trial are expected to improve the efficacy of prescriptive exercise training in subjects with non-specific chronic LBP.

The limitations of this study include the use of only two recruitment locations, which primarily represents the anglophone population of Montreal. This will decrease the generalizability of this study. We are also restricting inclusion to those able to understand and read English or French, which will also limit generalizability. Furthermore, true blinding of exercise supervisors/providers is not possible within an exercise trial. However, the participants will not be told their group assignment and only that they are completing an exercise protocol with the aim of decreasing their LBP and increasing their function.

## Data Availability

Not applicable.

## Abbreviations

LBP: Low back pain
ODI: Oswestry disability index for low back pain
GE: General exercise
MC + ILEX: Motor control and isolated lumbar extension exercise
